# Prediction of the Need for Anticonvulsants in the Management of Orofacial Neuropathic Pain Using Machine Learning

**DOI:** 10.7759/cureus.58934

**Published:** 2024-04-24

**Authors:** Ramya Suresh, Pradeep Kumar Yadalam, Ramya Ramadoss, Karthikeyan Ramalingam

**Affiliations:** 1 Oral Biology, Saveetha Dental College and Hospitals, Saveetha Institute of Medical and Technical Sciences, Saveetha University, Chennai, IND; 2 Periodontics, Saveetha Institute of Medical and Technical Sciences, Chennai, IND; 3 Oral Pathology and Microbiology, Saveetha Dental College and Hospitals, Saveetha Institute of Medical and Technical Sciences, Saveetha University, Chennai, IND

**Keywords:** neuralgia, artificial intelligence, machine learning, anticonvulsants, orofacial neuropathic pain

## Abstract

Background and aim

Orofacial neuropathic pain is a medical condition caused by a lesion or dysfunction of the nervous system and is one of the most challenging for dental clinicians to diagnose. Anticonvulsants, antidepressants, analgesics, nonsteroidal anti-inflammatory drugs, and other classes of medications are frequently used to treat this condition. Our study aimed to build a machine learning-based classifier to predict the need for anticonvulsant drugs in patients with orofacial neuropathic pain.

Materials and methods

A machine learning tool that was trained and tested on patients for predicting and detecting algorithms, which would in turn predict the need for anticonvulsants in the treatment of orofacial neuropathic pain, was employed in this study.

Results

Three machine learning algorithms successfully detected and predicted the need for anticonvulsants to treat patients with orofacial neuropathic pain. All three models showed a high accuracy, that is, 97%, 94%, and 89%, in predicting the need for anticonvulsants.

Conclusion

Machine learning algorithms can accurately predict the need for anticonvulsant drugs for treating orofacial neuropathic pain. Further research is needed to validate these findings using larger sample sizes and imaging modalities.

## Introduction

Orofacial neuropathic pain is a medical disorder induced by damage to the orofacial somatosensory nervous system. It may be spontaneous or a result of a local injury or systemic disease, making differential diagnosis challenging [1, 2]. By its primary fundamental cause, orofacial pain can generally be divided into three categories: somatic, neuropathic, and psychological. Several identified conditions such as periodontal and pulpal diseases, oral ulcers, Eagle's syndrome, burning mouth syndrome, headaches, temporomandibular joint disorders, oral cancer, and neuropathic pathologies can cause orofacial discomfort. Multiple forms of orofacial neuropathic pain including trigeminal neuralgia, atypical odontalgia, nonodontogenic neuropathic orofacial pain, postherpetic neuralgia, atypical odontalgia, and glossopharyngeal neuralgia have been identified [3]. Depending on the underlying cause, orofacial neuropathic pain may be persistent or episodic and other characteristic symptoms may vary substantially. Since dentists rarely consider neuropathic pain as a potential cause, differential diagnosis and early identification of orofacial neuropathic pain are essential to avoid unnecessary dental procedures. Although the pathophysiological mechanisms underlying orofacial neuropathic pain are not completely understood, it is believed to share several characteristics with other peripheral neuropathies.

A multidisciplinary approach involving psychological, pharmacological, and physiological therapy is necessary to manage orofacial neuropathic pain. Currently, pharmacological intervention is regarded as the cornerstone of neuropathic pain management. Anticonvulsants, antidepressants, analgesics, nonsteroidal anti-inflammatory drugs, and other classes of medications are frequently used to treat neuropathy. Among them, anticonvulsants have shown efficacy in treating orofacial neuropathy. Gabapentin is a commonly used medication that modulates calcium channels and reduces the neurotransmitter production of pain. Due to their efficacy and safety profiles, these medicines are frequently used as the initial treatment options. Carbamazepine and ox-carbamazepine are commonly used medications that block sodium channels and inhibit abnormal nerve discharge. These drugs are also used in the treatment of other medical conditions such as diabetes, cancer, and multiple sclerosis [4].

Machine learning brings several advantages when addressing the need for anticonvulsants in patients with pain. Firstly, it enables personalized treatment plans by analyzing diverse patient data and identifying patterns influencing treatment outcomes. This personalized approach improves the likelihood of successful pain management by tailoring treatment strategies to individual patients [5]. Additionally, machine learning models offer enhanced accuracy by considering multiple variables simultaneously and identifying subtle relationships that may not be apparent through traditional methods. This leads to more accurate predictions and recommendations, aiding healthcare providers in making informed decisions regarding anticonvulsant prescriptions.

Another advantage is the efficient allocation of resources. By identifying patients who are more likely to benefit from anticonvulsant treatment, machine learning models help optimize resource utilization. This ensures that limited resources, such as medications, are directed toward patients with a higher probability of positive treatment outcomes [6, 7]. Machine learning contributes to more efficient resource allocation in pain management by minimizing unnecessary treatments and associated costs. Overall, machine learning is a valuable decision-support tool, offering objective and evidence-based recommendations for anticonvulsant usage, improving treatment efficacy, and optimizing resource utilization in patients with pain. This study aims to contribute to this area of research by exploring the predictive accuracy of machine learning algorithms in the need for anticonvulsants for treating orofacial neuropathic pain patients.

## Materials and methods

The study received approval from the ethical committee of Saveetha Dental College and Hospitals. Orofacial neuralgia patients were selected from a database administered by dental information software at Saveetha Dental College and Hospitals. A cohort of 178 individuals, who were receiving anticonvulsant medication for orofacial neuropathic pain, were recruited based on the data in the Dental Information Archiving Software (DIAS). The gathered data was subjected to normalization and the elimination of outliers. Then, the procedure of data preparation and exploratory analysis was carried out. The dataset was partitioned into two categories: 80% for training, which served as the representative sample, and 20% for testing. The models were trained and evaluated using manual labeling by professionals as a reference [7, 8].

**Figure 1:**
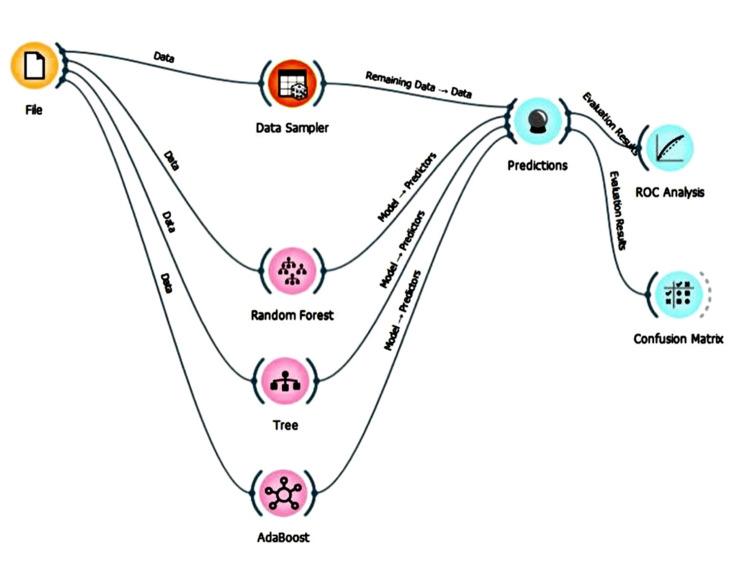
Workflow of the machine learning model with widgets procured from Orange (data mining software) ROC: receiver operating characteristic

The study was carried out using the Orange data mining tool. It is an open source software hosted under general public license (GPL) on GitHub and uses common Python open-source libraries for scientific computing (Figure 1). Models were constructed using the Orange artificial intelligence (AI) tool. Different algorithms like decision tree, AdaBoost, and neural network were used to predict the accuracy and validation using a confusion matrix. Precision and recall were also analyzed for this clinical study [9].

AdaBoost

AdaBoost, short for adaptive boosting, is an ensemble learning technique introduced by Freund and Schapire in 1997. It is widely used for binary classification problems and is considered one of the first foundational boosting algorithms. The core idea behind boosting algorithms is combining multiple weak learners (usually simple models) to create a single strong learner to make accurate predictions. The algorithm is designed to improve the prediction power of the model by iteratively focusing on the misclassified instances from previous models [10].

Decision tree

A decision tree is a supervised learning algorithm used in machine learning for regression tasks and classification tasks. It is a popular and intuitive algorithm representing a hierarchical, tree-like structure consisting of nodes and branches. The decision tree starts at the root node and branches into internal nodes based on specific conditions or features. Each internal node represents a decision based on a feature, and each branch represents the possible outcomes or values of that feature. The process continues recursively until the leaf nodes are reached, which are the final decision points or predictions.

Random Forest

Random Forest is a powerful ensemble machine learning algorithm for the classification and regression tasks. It is an extension of the decision tree algorithm and leverages the collective predictions of multiple decision trees to arrive at a final result [10].

Confusion matrix

A confusion matrix is a tabular representation utilized for assessing the efficacy of a classification algorithm through the comparison of predicted labels with the true labels presented in a given dataset. The provided information offers a comprehensive analysis of the true positives, true negatives, false positives, and false negatives, facilitating a more comprehensive assessment of the algorithm's performance.

Area under the curve (AUC)-receiver operating characteristic (ROC) curve

The classification models are evaluated using area under the curve-receiver operating characteristic (AUC-ROC) curve. The AUC-ROC metric can determine a model's ability to distinguish classes. Models with higher AUCs perform better. AUC-ROC curves are often used to graphically show the relationship and trade-off between sensitivity and specificity for every possible cut-off for a test or combination of tests. One way to assess model accuracy is using the AUC value. A good model has a separability AUC near 1. A model with a low AUC has the worst separability [11].

## Results

Orange data mining is a framework for data analysis with clustering, classification, and interactive data and model visualization components. Orange and the extensions are open source. Figure 1 shows the workflow of Orange with widgets. Orange data analysis workflows are defined by widget selection and connections.

**Figure 2:**
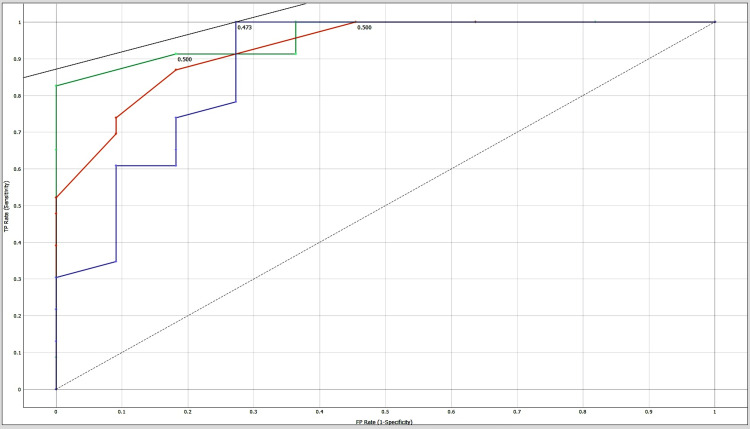
ROC curve for carbamazepine ROC: Receiver operating characteristic

**Figure 3:**
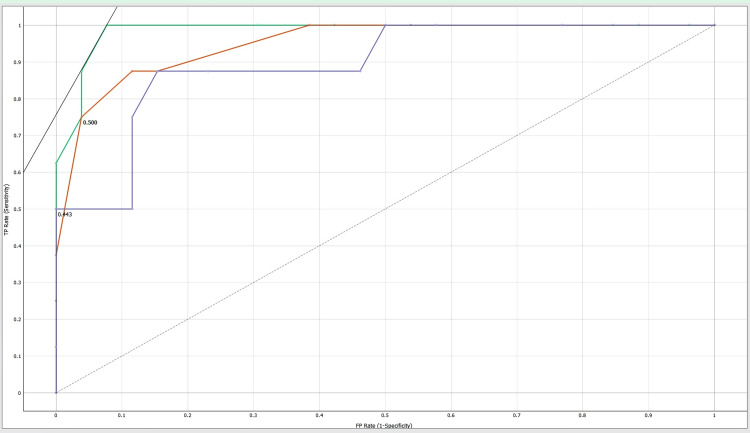
ROC curve for gabapentin ROC: receiver operating characteristic

**Figure 4:**
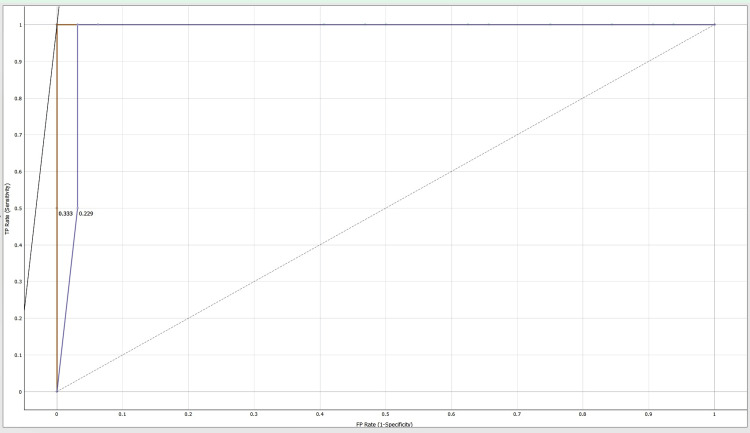
ROC curve for ox-carbamazepine ROC: receiver operating characteristic

Machine learning results show an accuracy of 97%, 94% and 89% as shown in Table 1. These results confirm that AdaBoost, decision tree, and Random Forest have good predictability. Tables 2-4 show the confusion matrixes for AdaBoost, decision tree, and Random Forest. Figures 2-4 show the ROC curves for carbamazepine, gabapentin, and ox-carbamazepine.

**Table 1:** Validation of test data for all the chosen algorithms with an AUC of 97%, 94%, and 89% AUC: Area under the curve AdaBoost: Algorithm for the classification and regression tasks CA: Class accuracy Decision tree: Decision tree algorithm used for regression tasks and classification tasks Random Forest: Algorithm for collective predictions of multiple decision trees

Model	AUC	CA	F1	Precision	Recall	Specificity
AdaBoost	0.975	0.824	0.816	0.845	0.824	0.692
Decision tree	0.944	0.765	0.715	0.754	0.765	0.569
Random Forest	0.897	0.824	0.791	0.791	0.824	0.692

 

**Table 2:** Confusion matrix for AdaBoost Confusion matrix: Tabular representation utilized for assessing the efficacy of a classification algorithm AdaBoost: Algorithm for the classification and regression tasks

	Carbamazepine	Carbamazepine, gabapentin	Carbamazepine, ox-carbamazepine	Gabapentin	Ox-carbamazepine	Σ
Carbamazepine	22	0	0	0	0	23
Carbamazepine, gabapentin	1	0	0	0	0	1
Carbamazepine, ox-carbamazepine	0	0	0	0	0	0
Gabapentin	3	1	0	5	0	8
Ox-carbamazepine	2	0	0	0	0	2
Σ	27	1	0	5	0	34

**Table 3:** Confusion matrix for the decision tree Decision tree: Decision tree algorithm used for regression tasks and classification tasks

	Carbamazepine	Carbamazepine, gabapentin	Carbamazepine, ox-carbamazepine	Gabapentin	Ox-carbamazepine	Σ
Carbamazepine	23	0	0	0	0	23
Carbamazepine, gabapentin	1	0	0	0	0	1
Carbamazepine, ox-carbamazepine	0	0	0	0	0	0
Gabapentin	4	1	0	3	0	8
Ox-carbamazepine	2	0	0	0	0	2
Σ	30	1	0	3	0	34

**Table 4:** Confusion matrix for the Random Forest Random Forest: Algorithm for collective predictions of multiple decision trees

	Carbamazepine	Carbamazepine, Gabapentin	Carbamazepine, ox-carbamazepine	Gabapentin	Ox-carbamazepine	Σ
Carbamazepine	23	0	0	0	0	23
Carbamazepine, Gabapentin	1	0	0	0	0	1
Carbamazepine, ox-carbamazepine	0	0	0	0	0	0
Gabapentin	2	1	0	5	0	8
Ox-Carbamazepine	2	0	0	0	0	2
Σ	28	1	0	5	0	34

## Discussion

Orofacial neuropathic pain is a severe disorder that requires proper diagnosis and management. It affects a person's quality of life and can be activated by touch, temperature changes, and movement. The underlying causes vary but often involve nerve dysfunction or injury. The commonly reported conditions include trigeminal neuralgia, glossopharyngeal neuralgia, postherpetic neuralgia, and periodic migrainous neuralgia. Pain is typically burning, shooting, or electric shock-like, accompanied by numbness, tingling, or altered sensations [12].

The current treatment approaches for orofacial neuropathic pain involve a multimodal approach that includes pharmacotherapy, physical therapies, and psychological interventions. Drugs from different classes, including anticonvulsants, antidepressants, opioids, and nonsteroidal anti-inflammatory drugs, are commonly used to treat orofacial neuropathy [13]. Topical agents such as lidocaine patches and capsaicin cream offer localized pain relief by numbing the affected area or desensitizing the nerve fibers. In severe cases, opioids are used but are limited due to the risk of dependence and potential side effects. Physical therapy includes jaw exercises that improve jaw muscle function, reduce muscle tension, and enhance jaw joint stability. Psychological interventions such as cognitive-behavioral therapy help patients to develop coping strategies, change negative thought patterns, and manage pain related to anxiety and depression. Anticonvulsants play a crucial role in the management of orofacial neuropathic pain. Medications such as gabapentin, carbamazepine, pregabalin, and ox-carbamazepine are widely used as they are effective in reducing the pain by stabilizing hyper-excitable nerves and modulating pain signal transmission in the central nervous system by targeting voltage-gated calcium channels and inhibiting excessive release of excitatory neurotransmitters. Anticonvulsants help normalize neuronal activity and alleviate orofacial neuropathic pain. They are often used in combination therapy, working synergistically with other medications to enhance pain relief. They are initially given at low doses and then titrated gradually to minimize side effects. While the efficacy of anticonvulsant drugs varies among individuals, these medications have demonstrated significant benefits in reducing pain intensity, improving sleep, and enhancing the overall quality of life for patients suffering from chronic orofacial neuropathic pain [14].

Machine learning techniques have revolutionized the field of drug discovery and prediction, offering valuable insights and accelerating the identification of potential therapeutic agents. Machine learning models can extract complex patterns and predict drug efficacy, toxicity, and personalized treatment by integrating and analyzing diverse datasets such as patients' clinical information. One significant application of machine learning in drug prediction is target identification. The machine learning models can identify potential targets associated with specific diseases by mining large-scale clinical data [15]. Furthermore, machine learning algorithms enable virtual screening and drug repurposing by leveraging the existing drug data to predict efficacy against new targets. This approach saves time and resources and increases the chances of discovering new therapeutic applications for the existing drugs. There are fledgling studies using machine learning that show potential in supporting clinicians in making management decisions to treat orofacial neuralgia. Machine learning models are trained by the given clinical data [16].

Our study retrieved a sample size of 178 orofacial neuralgia patients' clinical data, and a machine learning model using Orange software was used. Different algorithms like a decision tree, AdaBoost, and a neural network (Random Forest) were used to predict the accuracy and validation using a confusion matrix (Tables 2-4). Precision and recall were also analyzed for this clinical study. The ROC curve is a crucial tool in image analysis, as it shows the false-positive rate (FPR) and true-positive rate (TPR), with the curve shape indicating an increase in sensitivity with an increase in false-positive rate. Multiple curves represent different models or threshold settings and the area under the curve (AUC) quantifies test performance. Our predicted model showed an accuracy of 97% (Table 1), which is comparatively higher than that in the study conducted by Daniel Lopez-Martinez et al. [17] who reported a maximum accuracy of 72% in machine learning for pain assessment and management, concluding that the tailored model showed a good outcome. This prediction model will help clinicians and pain specialists in advising anti-convulsants for the treatment of orofacial pain.

Limitations

The study is subject to certain limitations. Accurately collecting all important parameters that impact therapy response is challenging due to the variability of neuropathic pain syndromes within the orofacial area. Pain perception and modulation include intricate interactions among biological, psychological, and social components, which may not be adequately captured by machine learning algorithms due to their multifactorial nature. Despite these limitations, machine learning exhibits a potential for augmenting personalized therapy methodologies for orofacial neuropathic pain by identifying predictive biomarkers and optimizing therapeutic strategies.

## Conclusions

In our study, we introduced a machine learning approach to predict the need for anticonvulsants in patients with orofacial neuropathic pain. Our predicted model had a high degree of accuracy of 97% in predicting the need for anticonvulsants. The insights generated by our model have significant value as a reference point for orofacial pain specialists and neurologists and can be of great assistance when deciding on treatment regimens. In addition, the predicted machine learning model is readily accessible as it utilizes standard computers and open-source software, making its adoption in hospitals a viable option.
